# Lipid phosphatase SHIP‐1 regulates chondrocyte hypertrophy and skeletal development

**DOI:** 10.1002/jcp.29063

**Published:** 2019-07-09

**Authors:** Eui‐Young So, Changqi Sun, Keith Q. Wu, Adam Driesman, Susan Leggett, Mauricio Isaac, Travis Spangler, Patrycja M. Dubielecka‐Szczerba, Anthony M. Reginato, Olin D. Liang

**Affiliations:** ^1^ Division of Hematology/Oncology, Department of Medicine, Rhode Island Hospital Warren Alpert Medical School of Brown University Providence Rhode Island; ^2^ Department of Orthopaedics, Rhode Island Hospital Warren Alpert Medical School of Brown University Providence Rhode Island; ^3^ Division of Rheumatology, Department of Medicine, Rhode Island Hospital Warren Alpert Medical School of Brown University Providence Rhode Island

**Keywords:** bone marrow microenvironment, chondrocyte hypertrophy, lipid phosphatase SHIP‐1, osteochondral progenitor cells, skeletal development

## Abstract

SH2‐containing inositol‐5′‐phosphatase‐1 (SHIP‐1) controls the phosphatidylinositol‐3′‐kinase (PI3K) initiated signaling pathway by limiting cell membrane recruitment and activation of Akt. Despite the fact that many of the growth factors important to cartilage development and functions are able to activate the PI3K signal transduction pathway, little is known about the role of PI3K signaling in chondrocyte biology and its contribution to mammalian skeletogenesis. Here, we report that the lipid phosphatase SHIP‐1 regulates chondrocyte hypertrophy and skeletal development through its expression in osteochondroprogenitor cells. Global SHIP‐1 knockout led to accelerated chondrocyte hypertrophy and premature formation of the secondary ossification center in the bones of postnatal mice. Drastically higher vascularization and greater number of c‐kit + progenitors associated with sinusoids in the bone marrow also indicated more advanced chondrocyte hypertrophic differentiation in SHIP‐1 knockout mice than in wild‐type mice. In corroboration with the in vivo phenotype, SHIP‐1 deficient PDGFRα + Sca‐1 + osteochondroprogenitor cells exhibited rapid differentiation into hypertrophic chondrocytes under chondrogenic culture conditions in vitro. Furthermore, SHIP‐1 deficiency inhibited hypoxia‐induced cellular activation of Akt and extracellular‐signal‐regulated kinase (Erk) and suppressed hypoxia‐induced cell proliferation. These results suggest that SHIP‐1 is required for hypoxia‐induced growth signaling under physiological hypoxia in the bone marrow. In conclusion, the lipid phosphatase SHIP‐1 regulates skeletal development by modulating chondrogenesis and the hypoxia response of the osteochondroprogenitors during endochondral bone formation.

## Graphical abstract

In this study, we found that lipid phosphatase SH2‐containing inositol‐5′‐phosphatase‐1 (SHIP‐1) negatively regulates the hypertrophic process of growth plate chondrocytes. SHIP‐1 deficiency accelerates the formation of the secondary ossification center, and SHIP‐1 KO mice have elevated vascularization and more c‐kit + progenitor cells in the bone marrow compartment. SHIP‐1 is also required for hypoxia‐induced Akt/Erk activation and cell proliferation.

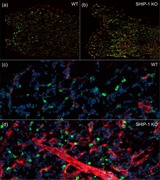

## 1 INTRODUCTION

Skeletal development begins in the embryo when multipotent mesenchymal cells arise from the ectoderm and mesoderm, migrate to specific sites and commit to a skeletal fate. While most skeletogenic progenitors will differentiate into chondrocytes to form cartilage and later into osteoblasts to form bone, some remain as mesenchymal stem cells (MSCs) in the bone marrow (BM) throughout life (Kobayashi & Kronenberg, 2014). The primary skeleton, which is entirely cartilaginous, will progressively transform into bone during fetal and postnatal growth through a developmental process known as endochondral ossification (Mackie, Ahmed, Tatarczuch, Chen, & Mirams, 2008). During endochondral bone formation, chondrocytes undergo proliferation, hypertrophic differentiation and apoptosis (Stevens & Williams, 1999). Blood vessel invasion is accompanied by an influx of osteoblastic progenitors from the perichondrium resulting in the replacement of cartilage with bone (Kronenberg, 2003; Long & Ornitz, 2013; Maes et al., 2010). A corollary of the complexity of the skeletal development is a high incidence of osteochondrodysplasia at a young age, which often constitutes high risk for skeleton degenerative diseases such as osteoarthritis and osteoporosis later in life (Lefebvre & Bhattaram, 2010; Long & Ornitz, 2013). Thus, elucidating the biological processes that control endochondral bone development is critical for understanding human skeletal dysplasia and adult abnormal fracture healing.

Despite the fact that many of the growth factors important to cartilage development and functions are able to activate the phosphatidylinositol‐3′‐kinase (PI3K) signal transduction pathway, little is known about the role of PI3K signaling in chondrocyte biology and its contribution to mammalian skeletogenesis (Guntur & Rosen, 2013; Kita, Kimura, Nakamura, Yoshikawa, & Nakano, 2008; Ulici et al., 2009). Specific ligand‐receptor interactions recruit PI3K into proximity with its substrate phosphatidylinositol (4,5)‐bisphosphate, PI(4,5)P2, to generate the second messenger phosphatidylinositol (3,4,5)‐trisphosphate, PI(3,4,5)P3. SH2‐containing inositol‐5′‐phosphatase‐1 (SHIP‐1) hydrolyzes PI(3,4,5)P3 to PI(3,4)P2. Both PI(3,4,5)P3 and PI(3,4)P2 have a significant affinity for pleckstrin homology domain‐containing kinases (e.g. Akt) that serve as effectors of PI3K signaling (Franke, Kaplan, Cantley, & Toker, 1997; Rohrschneider, Fuller, Wolf, Liu, & Lucas, 2000). Unlike the 3′‐phosphatase and tumor suppressor PTEN, which primarily controls the levels of PI(3,4,5)P3 (Song, Salmena, & Pandolfi, 2012), the 5′‐phosphatase SHIP‐1 modulates PI3K initiated signaling by limiting membrane recruitment and activation of Akt through both its substrate PI(3,4,5)P3 and its product PI(3,4)P2 (Fernandes, Iyer, & Kerr, 2013; Ma, Cheung, Marshall, & Duronio, 2008; Scheid et al., 2002). While inactivation of PTEN in osteochondroprogenitor cells results in epiphyseal growth plate abnormalities and skeletal overgrowth (Ford‐Hutchinson et al., 2007), global deletion of SHIP‐1 in mice leads to severe osteoporosis reportedly due to increased numbers of hyper‐resorptive osteoclasts (Takeshita et al., 2002). However, a recent report by Iyer, Viernes, Chisholm, Margulies, and Kerr (2014) demonstrated that mice with myeloid‐restricted ablation of SHIP‐1, including osteoclasts, show no reduction in bone mass or density. The study suggests that bone mass reduction of the knockout mice is mainly due to SHIP‐1 deficiency in MSCs and osteolineage progenitors. Previous studies by Hazen et al. (2009) show that SHIP‐1 is expressed by stromal cells and regulates their differentiation and support of the hematopoietic stem cells. Selective ablation of SHIP‐1 in MSCs causes the skeletal defects observed in germline null mice, further demonstrating that SHIP‐1 is expressed by MSCs and regulates their self‐renewal and differentiation (Iyer et al., 2014). We recently discovered that deficiency of the lipid phosphatase SHIP‐1 could enable long‐term reconstitution of the hematopoietic BM microenvironment (Liang et al., 2013). However, the mechanism with which SHIP‐1 is required for a functional hematopoietic niche remains unclear. Here, we report that SHIP‐1 plays a critical role in skeletal development, where in the absence of SHIP‐1, accelerated chondrocyte hypertrophy and increased vascularization leads to reduced skeletal size, altered bone architecture, and BM microenvironment.

## 2 MATERIALS AND METHODS

### 2.1 Mouse maintenance and genotyping

Animal procedures followed protocols approved by the Institutional Animal Care and Use Committees at Rhode Island Hospital. The SHIP‐1 KO mice (Inpp5d < tm1Dmt > /J, Stock Nr. 003534) of a mixed genetic background (129/C57BL/6) were purchased from the Jackson Laboratory (Bar Harbor, ME) and backcrossed with C57BL/6 mice (C57BL/6 J, Stock Nr. 000664) for multiple generations. Mouse genotyping was carried out following protocols from the Jackson Laboratory.

### 2.2 Histology and immunohistochemistry

For histological analyses, the hind limbs of the postnatal mice were dissected, fixed in 10% formalin solution in neutral buffer. Paraffin sections were prepared following standard procedures. For morphological evaluation, the sections were stained with Safranin‐O/Fast Green using 1% Safranin‐O solution with counterstaining by hematoxylin and 0.2% Fast Green. For collagen type X (Col X) immunohistochemical (IHC) staining, the sections were deparaffinized with xylene and rehydrated in a descending series of ethanol concentrations. Heat‐mediated antigen retrieval was performed using 1 x citrate buffer (pH 6.0). The deparaffinized and rehydrated sections were blocked and incubated with 1:150 dilution of the anti‐collagen type X antibody (purified mouse monoclonal X53, 14–9771‐82, ThermoFisher, Waltham, MA) overnight at 4°C. Slides were then incubated with the EnVision Dual Link System‐HRP solution (Dako, Santa Clara, CA) containing goat anti‐mouse and anti‐rabbit immunoglobulins conjugated to the peroxidase‐labeled polymer. Following chromogenic development, the slides were counterstained with Harri's hematoxylin. Images were taken by using a Nikon Eclipse E800 microscope equipped with a camera and SPOT software (SPOT Imaging, Sterling Heights, MI).

### 2.3 Immunofluorescent staining and laser‐scanning cytometry of BM sections

Laser‐scanning cytometry was performed as described earlier (Nombela‐Arrieta et al., 2013). Briefly, the mice were perfused post‐mortem with 10 ml paraformaldehyde‐lysine‐periodate (PLP) fixative through the vena cava to achieve rapid in situ fixation and optimal preservation of the BM tissue. Femoral bones were isolated, fixed in PLP for 4–8 hr, rehydrated in 30% sucrose/phosphate‐buffered saline (PBS) for 48 hr and snap frozen in OCT (TissueTek). Cryosections of non‐decalcified whole longitudinal femoral bones were obtained using a Leica Cryostat and the Cryojane tape transfer system (Leica Microsystems). BM sections were stained with rabbit anti‐Laminin (Sigma Aldrich; L9393) and goat anti‐c‐kit (R&D systems; AF1356) polyclonal antibodies. As secondary antibodies, DyLight 488‐donkey anti‐goat immunoglobulin G (IgG) and DyLight‐649 donkey anti‐rabbit IgG (Jackson Immunoresearch) were employed. 4′,6‐Diamidino‐2‐phenylindole (DAPI; Invitrogen) staining was used for nuclear detection and sections were mounted with Vectashield mounting medium for immunofluorescence (Vector Labs). High‐resolution images of whole longitudinal immunostained femoral sections were obtained with an iCys Research Imaging Cytometer (Compucyte Corporation) equipped with four laser lines (405, 488, 561, and 633 nm) and four PMT detectors with bandpass emission filters at 450/40, 521/15, 575/50, and 650LP.

### 2.4 PDGFRα + Sca‐1 + (PαS) MSC isolation and cultivation

Murine BM PαS MSCs from WT and SHIP‐1 KO mice (6‐week old) were isolated and cultured using reagents and methods as previously described by Morikawa et al. (2009). Briefly, femurs and tibias from WT or SHIP‐1 KO mice were dissected and crushed with mortar and pestle. The bone fragments were gently washed once in HBSS supplemented with 2% fetal bovine serum (FBS), 10 mM Hepes, and 1% penicillin/streptomycin (P/S). The cell suspension filtered through a 70 μm cell strainer (BD Falcon) was discarded. The bone fragments were incubated for 1 hr at 37°C in 20 ml DMEM (Invitrogen) containing 0.2% collagenase (Wako Chemicals), 10 mM Hepes, and 1% P/S. The cell suspension was filtered through a cell strainer to remove debris and bone pieces, and cells were collected by centrifugation at 280*g* for 7 min at 4°C. The pellet was immersed in 1 ml water for 5–10 s to burst the red blood cells, after which 1 ml of 2 × PBS containing 4% FBS was added, and the suspension was filtered through a cell strainer. The cells were suspended in ice‐cold HBSS containing supplements as above at 1–5 × 10^7^ cell/ml, and stained for 30 min on ice with the following antibodies APC‐PDGFRα (APA5), FITC‐Sca‐1 (Ly6A/E), PE‐CD45 (30‐F11), and PE‐Ter119 (Ter‐119) (all from eBioscience). Flow cytometry analysis and sorting were performed on a Beckman Coulter MoFlo Legacy with software Summit version 4.3. The CD45‐, Ter119‐, PDGFRα+, and Sca‐1+ (PαS) cells were allowed to adhere to the plastic surface of a 25 cm^2^ tissue culture flask (Falcon 3081) for 48 hr without disturbance in α‐MEM medium (Invitrogen) supplemented with 10% nonheat‐inactivated FBS (Hyclone), 10% horse serum (Sigma), 1x l‐Glutamine (Invitrogen) and 1% P/S (Peister et al., 2004).

### 2.5 PαS MSC proliferation assay

Proliferation of PαS MSCs was measured using a 3‐(4,5‐demethylthiazol‐2‐yl)‐2,5‐diphenyltetrazolium bromide (MTT) assay kit (Cayman Chemical) according to the manufacturer's instruction. In brief, the cells were seeded at a density of 5 × 10^3^ per well in a 96‐well plate in 100 μl of complete medium in a regular CO_2_ incubator or in a hypoxia chamber. At the indicated time points, 100 μl MTT reagent was added into each well, and then formazan crystals were extracted by crystal dissolving solution. Absorbance was measured with a microplate reader at 570 nm (Molecular Devices).

### 2.6 Sodium dodecyl sulfate polyacrylamide gel electrophoresis (SDS‐PAGE) and immunoblotting

PαS MSC total cell lysates were prepared in M‐PER lysis buffer (Thermo Fisher Scientific) plus protease inhibitor cocktail (Halt, Thermo Fisher Scientific), and separated by using an 8.0% SDS polyacrylamide gel. Protein transfer onto a polyvinylidene difluoride (PVDF) membrane (Immobilon‐P, Millipore) was carried out in a semidry transfer unit (Trans‐Blot, Bio‐Rad) in 25 mM Tris, 192 mM Glycine, and 20% methanol for 30 min to 1 hr at 20 V. Membranes were blocked in 5% nonfat dried milk in Tris‐buffered saline (TBS)/0.1% Tween‐20 and incubated with primary antibodies and then fluorescence‐labeled secondary antibodies (LI‐COR Biotechnology), followed by scanning on a fluorescence image reader (Odyssey, LI‐COR Biotechnology). Primary antibodies used in this study were anti‐Akt, anti‐Erk (Cell Signalling Technology), and anti‐hypoxia‐inducible factor‐1α (anti‐HIF‐1α; Cayman Chemical). Specific anti‐phosphorylation antibodies were used against phospho‐Akt (Ser473) and phospho‐Erk (Thr202/Tyr204) (Cell Signaling). Anti‐β‐actin antibody (Novus) was used to detect β‐actin as loading controls.

### 2.7 Chondrogenic differentiation of PαS MSCs

Chondrogenic differentiation of PαS cells was carried out using Mouse StemXVivo Base Media and Chondrogenic Supplement according to the manufacturer's recommendation (CCM005 and CCM006; R&D Systems). Briefly, approximately 2.5 × 10^5^ PαS MSCs were resuspended in 5 ml of the pre‐warmed completed StemXVivo Base Media. The cells were centrifuged at 200*g* for 5 m at room temperature, followed by aspiration of the media and resuspension of the cells in 0.5 ml of pre‐warmed completed StemXVivo Chondrogenic Differentiation Media. The cells were then spun down again and the cell pellets were allowed to incubate upright with the chondrogenic differentiation medium at 37°C and 5% CO_2_ for 21 days, with fresh medium every 3 days. The chondrogenic pellets were then fixed with 10% formalin (Sigma), paraffin embedded, and sectioned for hematoxylin and eosin (H&E) staining and immunohistochemistry. Chondrocyte differentiation was verified by using a sheep anti‐mouse collagen type II (Col II) polyclonal antibody (AF3615; R&D Systems), which was then visualized by using a NorthernLights 557‐conjugated Donkey Anti‐Sheep Secondary Antibody (NL010; R&D Systems). Hypertrophic chondrocyte differentiation was verified by using a mouse anti‐collagen type X (Col X) monoclonal antibody (X53) conjugated with eFluor 570, (41–9771‐82; Thermo Fisher Scientific). The nuclei were counterstained with DAPI (Biolegend). Images were taken with a Nikon Eclipse E800 fluorescence microscope equipped with a camera and SPOT software.

### 2.8 Reverse transcription and quantitative real‐time polymerase chain reaction (PCR)

Total RNA was purified from PαS MSCs using TRIzole reagent (Invitrogen). First‐strand complementart DNA (cDNA) was synthesized by using a Super Script III kit (Invitrogen) from 1 to 4 μg total RNA, according to the manufacturer's instructions. Forward and reverse primers were as follows: *Col2a1* (5′‐GCA GAA TGG GCA GAG GTA TAA‐3′ and 5′‐CTG ATA TCT CCA GGT TCT CCT TTC‐3′); *Col10a1* (5′‐AGC AAG GAC GAG AAG GTA TTG‐3′ and 5′‐GGT TCC CTG GGA TTC CTT TAG‐3′); *Vegfa* (5′‐CAG GCT GCT GTA ACG ATG AA‐3′ and 5′‐TCT GCG GAT CTT GGA CAA AC‐3′); *Kitl* (5′‐CGG GAA TCC TGT GAC TGA TAA T‐3′ and 5′‐GGA TCT AGT TTC TGG CCT CTT C‐3′); *Hgf* (5′‐CAA CAG TAG GGT GGA TGG TTA G‐3′ and 5′‐TGA GCC TTC AGG ACC ATA GA‐3′); *Bmp4* (5′‐GCG GTC CAG GAA GAA GAA TAA‐3′ and 5′‐CCT CTA CCA CCA TCT CCT GAT A‐3′); and *GAPDH* (5′‐GGA GAA ACC TGC CAA GTA TGA‐3′ and 5′‐CCT GTT GCT GTA GCC GTA TT‐3′). For quantitative analysis of gene expression, real‐time PCR was performed by using iQ SYBR Green Supermix in an iCycler (Bio‐Rad). *GAPDH* expression was assessed as an internal reference for quantification. Relative gene expression level was expressed by the fold increase over internal control samples. Every real‐time PCR was performed in triplicate.

### 2.9 Gene expression analysis of SHIP‐1 KO PαS MSCs

To determine the gene expression pattern of WT and SHIP‐1 KO PαS MSCs, we obtained the mouse MSC RT^2^ Profiler PCR Array (Qiagen), which allows simultaneous analysis of 84 key genes involved in maintenance, self‐renewal and differentiation of MSCs. Briefly, total RNA was prepared from WT and SHIP‐1 KO PαS MSCs after 48 hr culture in hypoxia of 2% oxygen. Each cDNA was synthesized and mixed with RT^2^ SYBR Green qPCR mastermix (Qiagen) and an equal volume was added into each well of a 96‐well array plate. Real‐time PCR was performed using the CFX 96 Real‐Time System (Bio‐Rad). The *C*_t_ values for all wells were exported to a Microsoft Excel spreadsheet for use with the PCR Array Data Analysis software (Qiagen, http://www.SABiosciences.com/pcrarraydataanalysis.php) according to the manufacturer's instruction. The resulting data were shown by fold change that represents relative mRNA expression for SHIP‐1 KO PαS MSCs compared with WT cells.

### 2.10 Statistical analysis

Data are shown as the mean values ± *SEM*. The significance of difference in the mean values was calculated with an unpaired two‐tailed Student's *t* test using Microsoft Excel. *P* values of < .05 were considered to be statistically significant.

## 3 RESULTS

### 3.1 SHIP‐1 deficiency accelerates postnatal chondrocyte hypertrophy and secondary ossification center formation

**Figure 1:**
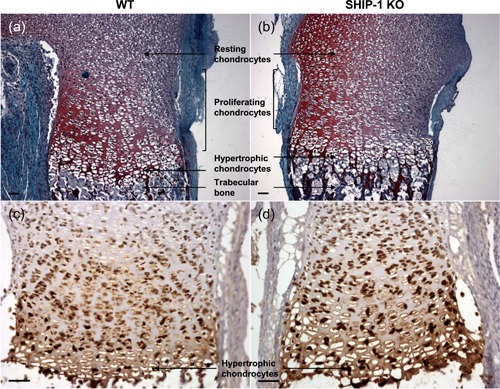
SHIP‐1 deficiency accelerates postnatal chondrocyte hypertrophy. (a and b) Safranin‐O/Fast Green staining of femoral cartilage sections of 1‐day old mice revealed that, while the growth plates in WT neonatal mice (a) (*n* = 5) had typical chondrocyte layers consist of the resting, proliferating, and hypertrophy zones, SHIP‐1 KO neonatal mice (b) (*n* = 5) appeared to have a more rapid transition from the resting chondrocyte to the hypertrophy chondrocyte layer. (c and d) Immunohistochemical staining of the hypertrophic chondrocyte marker collagen type X (brown spots) showed that, unlike the WT (c) (*n* = 5), SHIP‐1 KO (d) (*n* = 5) neonates had numerous hypertrophic chondrocytes throughout the growth plate. These hypertrophic chondrocytes in SHIP‐KO mice also appear earlier and larger in the growth plate than those in WT mice. Magnification: ×100 for (a) and (b); ×200 for (c) and (d). Scale bar = 50 μm. SHIP‐1, SH2‐containing inositol‐5′‐phosphatase‐1; WT, wild‐type [Color figure can be viewed at wileyonlinelibrary.com]

**Figure 2:**
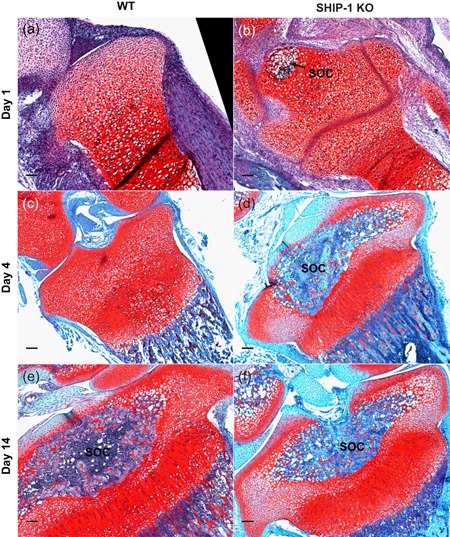
SHIP‐1 deficiency accelerates formation of the secondary ossification center. (a and b) Safranin‐O/Fast Green staining of the proximal tibia epiphysis of a WT (a) or a SHIP‐1 KO mouse (b) at postnatal Day 1 (*n* = 5 each). (c and d) Safranin‐O/Fast Green staining of the proximal tibia epiphysis of a WT (c) or a SHIP‐1 KO mouse (d) at postnatal Day 4 (*n* = 5 each). (e and f) Safranin‐O/Fast Green staining of the proximal tibia epiphysis of a WT (e) or a SHIP‐1 KO mouse (f) at postnatal Day 14 (*n* = 5 each). Magnification, ×100. Scale bar = 50 μm. SHIP‐1, SH2‐containing inositol‐5′‐phosphatase‐1; SOC, secondary ossification center; WT, wild‐type [Color figure can be viewed at wileyonlinelibrary.com]

To determine whether SHIP‐1 deficiency affects chondrocyte differentiation during endochondral ossification, skeletons from WT and SHIP‐1 KO neonatal mouse littermates were examined. At postnatal Day 1, no apparent skeletal defect could be seen per X‐ray imagining (data not shown). However, Safranin‐O/Fast Green staining of femoral cartilage sections of 1‐day old mice revealed that while the growth plates in WT neonatal mice displayed typical chondrocyte layers consisting of the resting, proliferating, and hypertrophy zones, SHIP‐1 KO neonatal mice appeared to have a more rapid transition from the resting chondrocyte to hypertrophy chondrocyte layer (Figure 1a,b). In support of this notion, immunohistochemical staining of the hypertrophic chondrocyte marker collagen type X (brown spots in Figure 1c,d) showed that, unlike the WT, SHIP‐1 KO neonates had numerous hypertrophic chondrocytes throughout the growth plate. These collagen type X positive cells in SHIP‐1 KO mice suggest that developmentally the hypertrophic chondrocytes appear earlier than those in WT mice. Furthermore, premature initiation of the secondary ossification center (SOC) had already begun at postnatal Day 1 in the proximal tibia epiphysis of SHIP‐1 KO but not in the WT mice (Figure 2a,b). At postnatal Day 4, formation of the SOC appeared to have already completed in the SHIP‐1 KO mice whereas the tibia epiphysis of the WT mice remained cartilaginous (Figure 2c,d). At postnatal Day 14, the SOC of the WT mice became established, which was about 10 days later than that in the SHIP‐1 KO mice (Figure 2e,f). These results indicate that SHIP‐1 deficiency accelerates both postnatal chondrocyte hypertrophy and secondary ossification center formation.

### 3.2 Defective skeleton and BM microenvironment in adult mice deficient of SHIP‐1

**Figure 3:**
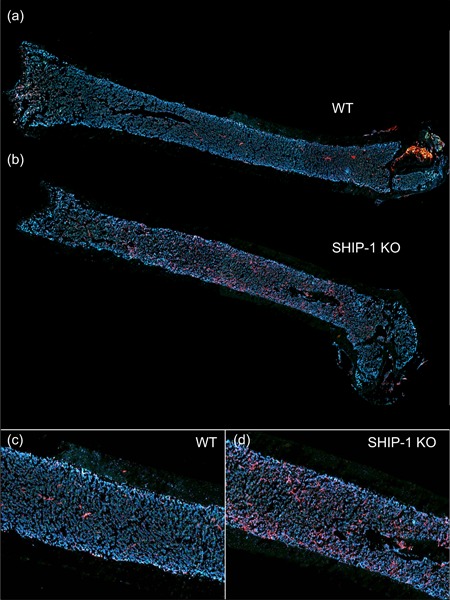
SHIP‐1 deficiency causes drastically increased vascularization in bone marrow. (a and b) Laser‐scanning cytometry imaging (LSC) of WT (a) and SHIP‐1 KO (b) femurs (*n* = 5 each). (c and d) Higher magnification of diaphysis section of WT (c) and SHIP‐1 KO (d) femurs. The vasculature was stained with rabbit anti‐laminin polyclonal antibody and was visualized in red with DyLight 549 goat anti‐rabbit IgG secondary antibody. The nuclei were counterstained with DAPI in blue. DAPI, 4′,6‐diamidino‐2‐phenylindole; IgG, immunoglobulin G; LSC, laser‐scanning cytometry; SHIP‐1, SH2‐containing inositol‐5′‐phosphatase‐1; WT, wild‐type [Color figure can be viewed at wileyonlinelibrary.com]

**Figure 4:**
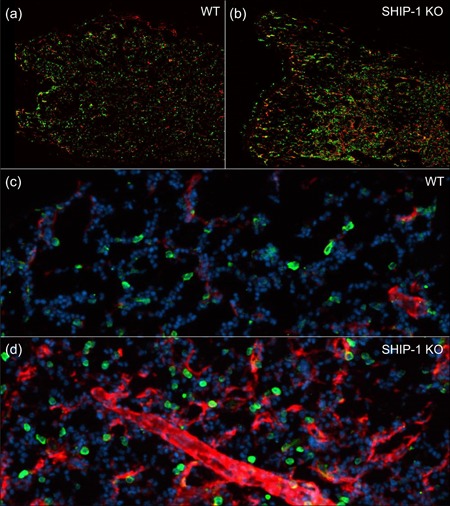
Increased progenitor cell frequency associated with enhanced vessel density in SHIP‐1 KO bone marrow. (a and b) LSC of immunofluorescence labeled femoral diaphysis of WT (a) and SHIP‐1 KO (b) femurs (*n* = 5 each). (c and d) Higher magnification of diaphysis section of WT (c) and SHIP‐1 KO (d) femurs. The vasculature was stained with rabbit anti‐laminin polyclonal antibody and was visualized in red with DyLight 549 goat anti‐rabbit IgG secondary antibody. C‐kit + progenitors in green fluorescence were stained with goat anti‐c‐kit antibodies and donkey anti‐goat DyLight 488. The nuclei were counterstained with DAPI in blue. DAPI, 4′,6‐diamidino‐2‐phenylindole; IgG, immunoglobulin G; LSC, laser‐scanning cytometry; SHIP‐1, SH2‐containing inositol‐5′‐phosphatase‐1; WT, wild‐type [Color figure can be viewed at wileyonlinelibrary.com]

Since terminally differentiated hypertrophic chondrocytes effectively attract blood vessel invasion and co‐migrating osteoprogenitors from the perichondrium, we postulate that premature chondrocyte hypertrophy would contribute to an altered BM microenvironment in the SHIP‐1 KO mice. Global SHIP‐1 deletion led to a smaller skeletal stature in adult mice (Figure S1a). H&E staining of the distal femoral epiphysis revealed that SHIP‐1 KO mice had thinner femoral cortical bones, augmented, thickened and disorganized trabeculae in a plate‐like pattern rather than the rod‐like pattern in WT mice (Figure S1b,c). To further determine the effect of SHIP‐1 deficiency on vascularization of the bones, we performed laser‐scanning cytometry (LSC) on femurs from both WT (Figure 3a,c) and SHIP‐1 KO mice (Figure 3b,d). The apparent volume of the blood vessel as visualized by laminin staining in red was significantly higher in the SHIP‐1 KO femur than in WT. Next, we examined whether the augmented vessel density in SHIP‐1 KO BM may increase the influx of progenitor cells including osteochondroprogenitors. As shown in Figure 4, a large number of c‐kit + progenitor cells were associated with sinusoidal vessels in SHIP‐1 deficient BM (Figure 4b,d) compared to WT BM (Figure 4a,c). This femoral marrow phenotype of the SHIP‐1 KO adult mice is reminiscent of vascular invasion forming primary spongiosa during the development of the primary ossification center, where osteoblastic progenitors become trabecular bone. Combined, these results indicate that SHIP‐1 plays a critical role in bone development through controlling chondrocyte hypertrophy, blood vessel invasion and density of trabecular bones.

### 3.3 Accelerated chondrocyte hypertrophy by SHIP‐1 KO MSCs in vitro

**Figure 5:**
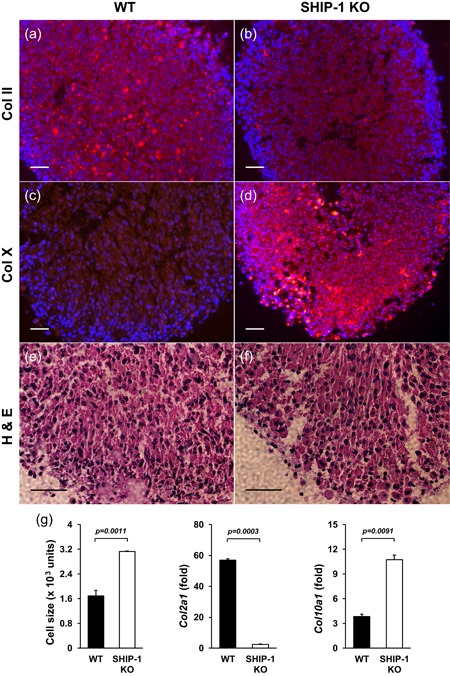
Accelerated chondrogenesis in SHIP‐1 KO PαS MSCs. PαS MSC pellets differentiated in vitro for 21 days using the Mouse StemXVivo Chondrogenic Base Media and Supplement were fixed, paraffin embedded, sectioned and IHC‐stained. (a and b) Sections from WT (a) and SHIP‐1 KO (b) PαS MSC pellets were stained with an antibody against the early chondrogenic lineage marker Col II. Bound antibody was visualized in bright red with a NorthernLights 557‐conjugated anti‐sheep secondary antibody. (c and d) Sections from WT (c) and SHIP‐1 KO (d) PαS MSC pellets were stained with a bright red eFluor570‐conjugated antibody against the hypertrophic chondrocyte lineage marker Col X. (e and f) Sequential sections from the same WT cell pellet as in (c) and from the same SHIP‐1 KO cell pellet as in (d) were subjected to H&E staining. Magnification: ×200 (a–d); ×400 (e and f). Scale bar = 50 μm. (g) Left panel, the difference in cell sizes between WT and SHIP‐1 KO PαS MSCs after chondrogenic induction was quantified by using the NIH ImageJ program and indicated as ImageJ Area Units. Middle and right panels, gene expression levels of *Col2a1* and *Col10a1* were evaluated by quantitative real‐time PCR. *GAPDH* expression was assessed as an internal reference for quantification. Gene expression levels were expressed as relative fold increase over the internal control. Student's *t* test was performed and *p* < .05 is considered significant. Col II, collagen type II; Col X, collagen type X; *GAPDH*, glyceraldehyde 3‐phosphate dehydrogenase; H&E, hematoxylin and eosin; IHC, immunohistochemical; MSC, mesenchymal stem cell; PCR, polymerase chain reaction; SHIP‐1, SH2‐containing inositol‐5′‐phosphatase‐1; WT, wild‐type [Color figure can be viewed at wileyonlinelibrary.com]

The PDGFRα + Sca‐1 + (PαS) MSCs were identified as a relatively homogenous subset of MSCs from the BM, and they can differentiate into osteoblasts, reticular cells and adipocytes in vivo once transplanted into irradiated mice (Morikawa et al., 2009). A recent study suggests that the early osteochondroprogenitor marker Prx1 labels the PαS MSC population that is required for hematopoietic stem cell maintenance by producing CXCL12 and ANG‐1 (Greenbaum et al., 2013). Given these characteristics, we chose to study the PαS MSCs as a model osteochondroprogenitor cell system in skeletal development and the BM microenvironment. We adopted the technique reported by Morikawa et al. (2009) to isolate the bone marrow PαS MSCs from WT and SHIP‐1 KO mice. Our previous characterization suggested that, compared to WT cells, SHIP‐1 KO PαS MSCs form fewer adipocytes and also appear to have impaired ability to form an osteoblastic nodule upon osteogenic induction (Liang et al., 2013). Here, in vitro chondrogenic differentiation was performed on these MSCs. After 21 days of chondrogenic induction, the WT PαS MSC pellet contained numerous Col II‐producing chondrocytes (bright red spots in Figure 5a), whereas SHIP‐1 KO MSC pellets had very few Col II‐positive cells (bright red spots in Figure 5b). In contrast, SHIP‐1 KO PαS MSCs produced significant numbers of Col X‐positive chondrocytes (bright red spots in Figure 5d), whereas WT PαS MSCs had none (Figure 5c). Both MSCs without chondrogenic induction were used as negative controls and showed no staining of both collagen antibodies (data not shown). Furthermore, sequential sections from the same cell pellets as in Figure 5c,d were subjected to H&E staining. No appreciable large hypertrophic cells were seen in WT MSC pellets (Figure 5e), but a number of large‐size hypertrophic cells could be seen in Figure 5f, corresponding to the area that stained positive for Col X in Figure 5d. The difference in cell sizes between WT and SHIP‐1 KO PαS MSCs after chondrogenic induction was quantified in Figure 5g. In addition, we performed real‐time PCR to quantify gene expression of *Col2a1* and *Col10a1*. Without the chondrogenic induction, SHIP‐1 KO PαS MSCs showed significantly higher expression of *Col10a1* gene but significantly lower expression of *Col2a1* gene than WT MSCs (Figure 5g). These in vitro data indicate that PαS MSCs from SHIP‐1 KO mice have intrinsically enhanced chondrocyte hypertrophic differentiation potential compared to WT mice.

### 3.4 SHIP‐1 deficiency abrogates hypoxia‐induced MSC proliferation

SHIP‐1 was shown to regulate neutrophil cell polarity, and loss of SHIP‐1 enhances neutrophil adhesion, which impairs chemotaxis (Mondal, Subramanian, Sakai, Bajrami, & Luo, 2012). Similarly, we observed reduced polarity in SHIP‐1 KO PαS MSCs. WT PαS MSCs displayed mainly an elongated and spindle‐shaped morphology at any stage of in vitro culture, but SHIP‐1 KO PαS cells showed a rounded and slightly elongated phenotype (Figure S2a). To evaluate if reduced polarity in the SHIP‐1 KO MSCs may affect cell proliferation, PαS MSCs were cultured under normoxia (Nx) or hypoxia (Hx) of 2% oxygen for 6 days. Cell proliferation was determined by using MTT assay each day. Hx significantly enhanced proliferation of WT PαS MSCs, but SHIP‐1 deficiency abrogated Hx‐induced proliferation (Figure S2b). These results suggest that SHIP‐1 may have a role in regulating MSC growth in the BM depending on oxygen concentration in the microenvironment.

### 3.5 Impaired cellular response to hypoxia by SHIP‐1 KO PαS MSCs

**Figure 6:**
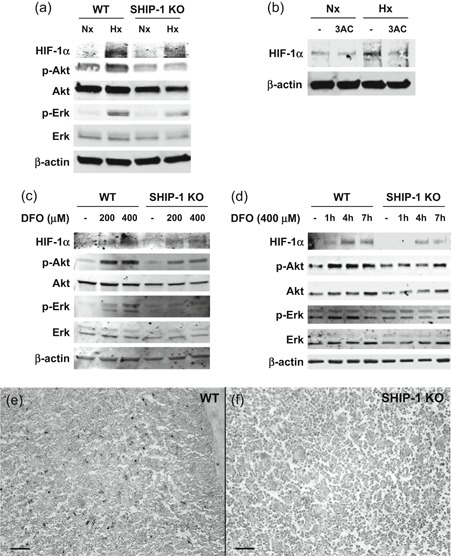
Impaired cellular response to hypoxia and hypoxia‐induced growth signaling by SHIP‐1 KO PαS MSCs. (a) Diminished HIF‐1α protein level and cellular activation of Akt and Erk in SHIP‐1 KO cells under Hx‐induction. WT and SHIP‐1 KO PαS MSCs were cultured under Nx or Hx for 24 hr. (b) Lack of increase in HIF‐1α protein level under Hx in WT cells with SHIP‐1 inhibition. WT PαS MSCs were incubated with a small molecule inhibitor of SHIP‐1 3AC at 10 μM for 3 hr before Hx treatment. (c and d) Diminished activation of Akt and Erk in SHIP‐1 KO cells. MSCs were cultured in the presence of a hypoxia‐mimicking chemical DFO (200 or 400 μM) for 24 hr (c) or treated with DFO at 400 μM for 1, 4, or 7 hr (d). Total protein extracts were prepared and subjected to western blot analysis using the indicated antibodies, and β‐actin was used as an internal loading control. (e and f) Immunohistochemical (IHC) staining of phosphorylated Akt in cells of WT and SHIP‐1 KO BM was performed, and plenty of IHC staining signals (black dots) could be visualized in the WT BM but none in the SHIP‐1 KO BM. Magnification: ×200 (e and f). Scale bar = 50 μm. BM, bone marrow; DFO, deferoxamine; Erk, extracellular‐signal‐regulated kinase; HIF‐1α, hypoxia‐inducible factor‐1α; Hx, hypoxia; IHC, immunohistochemical; MSC, mesenchymal stem cell; Nx, normoxia; SHIP‐1, SH2‐containing inositol‐5′‐phosphatase‐1; WT, wild‐type

MSCs reside in perivascular niches in the BM and in response to hypoxia express a number of genes associated with cell proliferation and survival (Mohyeldin, Garzon‐Muvdi, & Quinones‐Hinojosa, 2010; Ohnishi, Yasuda, Kitamura, & Nagaya, 2007; Spencer et al., 2014). Since SHIP‐1 deficiency abrogates hypoxia‐induced MSC proliferation, we examined protein expression of HIF‐1α in PαS MSCs cultured under Nx or Hx conditions. It has been well established that hypoxia (<5% O_2_) stabilizes the HIF‐1α protein, which is critical for osteogenesis and joint development (Araldi & Schipani, 2010). We found hypoxia‐induced HIF‐1α stabilization decreased in SHIP‐1 KO MSCs (Figure 6a). Furthermore, treatment with a selective SHIP‐1 inhibitor 3α‐aminocholestane (3AC) (Brooks et al., 2010), also suppressed hypoxia‐induced stabilization of HIF‐1α protein in WT MSCs (Figure 6b), suggesting SHIP‐1 is a key factor for cellular response to hypoxia in MSCs.

### 3.6 SHIP‐1 is required for hypoxia‐induced growth signaling in MSCs

It has been reported that SHIP‐1 activity is required to suppress PIP3‐induced Akt activation in multiple cell types, including neutrophils and MSCs, and SHIP‐1 regulates osteolineage commitment of MSCs through limiting induction of the PI3K/Akt/Id2 signaling pathway (Iyer et al., 2014; Mondal et al., 2012). Here, we investigate whether SHIP‐1 contributes to the activation of growth signaling in MSCs. WT and SHIP‐1 KO MSCs were cultured under normoxia or hypoxia (Figure 6a), or in the presence of deferoxamine (DFO), a hypoxia‐mimicking chemical (Figure 6c,d). The expression of Akt, Erk and their activation (phosphorylation) status were examined by immunoblotting. We found less induction of Akt phosphorylation in SHIP‐1 KO than in WT cells after either hypoxia or DFO treatment at different concentrations and time points. This finding was further supported by IHC staining of phosphorylated Akt in cells of WT and SHIP‐1 KO BM, where plenty of IHC staining signals (black spots) could be visualized in the WT BM but none in the SHIP‐1 KO BM (Figures 6e,f and S3a,b). Similarly, significantly reduced phosphorylation of Erk was also detected in SHIP‐1 KO PαS MSCs after either hypoxia or DFO treatment in vitro. Both Akt and Erk phosphorylation were well‐established as a proliferative response to growth factors and stresses in many cell types (Zhang & Liu, 2002). These results suggest that reduced stability of HIF‐1α combined with impaired activation of both Akt and Erk contributed to the decreased proliferative potential of SHIP‐1 KO PαS MSCs in the hypoxic BM.

### 3.7 Differential gene expression in WT and SHIP‐1 KO PαS MSCs under hypoxia

In addition, we also performed gene expression profiling on SHIP‐1 KO PαS MSCs and compared the expression levels of 84 key genes with those of the WT PαS MSCs after these cells were exposed to hypoxia treatment for 48 hr. The heat map generated by the Qiagen PCR Array Data Analysis software shows a fold change that represents relative mRNA expression for SHIP‐1 KO PαS MSCs compared with WT cells (Figure S4a). Significantly upregulated genes in SHIP‐1 KO MSCs included *Anpep* (29.5‐fold), *Alcam* (4‐fold), *Mmp2* (4‐fold), and *Vcam1* (3.5‐fold), which are involved in cell adhesion and migration, supporting morphological changes in SHIP‐1 KO MSCs. On the other hand, the expression of *Hgf* and *Tgfb3*, which are involved in growth signal activation, were markedly reduced in SHIP1‐KO compared to WT MSCs (Figure S4b). RT‐PCR was also performed to confirm comparable gene expression levels of *Vegfa* in both SHIP‐1 KO and WT PαS MSCs, and reduced gene expression levels of *Kitl*, *Hgf* and *Bmp4* in SHIP‐1 KO in comparison with WT PαS MSCs (Figure S4c).

## 4 DISCUSSION

Much of the mammalian skeleton is composed of bones that originate from cartilage templates through the process of endochondral bone formation. The transition from cartilage to bone is tightly coupled with chondrocyte proliferation, hypertrophy, blood vessel invasion and osteoblast differentiation in a temporal and spatial specific manner (Aguila & Rowe, 2005; Kobayashi & Kronenberg, 2014; Kronenberg, 2003; Maes, Carmeliet, & Schipani, 2012). During limb formation, for example, mesenchymal cells from the mesoderm condense and differentiate into cartilage anlagen surrounded by the perichondrium. The chondrocytes of the anlagen proliferate, and centrally placed cells exit the cell cycle, differentiating and undergoing hypertrophy. These events are characterized by a downregulation in collagen type II and upregulation of collagen type X. Indeed, SHIP‐1 KO PαS MSCs had low level *Col2a1* gene expression and high‐level *Col10a1* gene expression, indicating intrinsic hypertrophic differentiation potential.

Unlike the 3′‐phosphatase and tumor suppressor PTEN, which primarily controls the levels of PI(3,4,5)P3 (Song et al., 2012), the 5′‐phosphatase SHIP‐1 modulates PI3K initiated signaling by limiting membrane recruitment and activation of Akt through both its substrate PI(3,4,5)P3 and its product PI(3,4)P2 (Fernandes et al., 2013; Ma et al., 2008; Scheid et al., 2002). While PI(3,4,5)P3 and PI(3,4)P2 levels correlate with Akt phosphorylation at Thr308 and Ser473, respectively, PI(3,4)P2 is required and its levels determine Akt activity. Thus, SHIP‐1 deficiency may lead to lower levels of its product PI(3,4)P2 and less Akt activity in the bone marrow. Indeed, we could demonstrate this both in MSCs from SHIP‐1 KO mice as well as in immunohistochemical staining, which showed detectable phosphorylated Akt in WT but not in the SHIP‐1 KO mouse bone marrow. Based on these findings, increased numbers of c‐kit cells in the SHIP‐1 KO bone marrow may not necessarily be the result of phospho‐Akt driven proliferation. Instead, we postulate that the influx of these progenitor cells is due to enhanced vessel invasion, which follows chondrocyte hypertrophy.

Since chondrocyte hypertrophic differentiation precedes blood vessel invasion and osteoprogenitor influx (Maes et al., 2010; Sacchetti et al., 2007), accelerated differentiation into hypertrophic chondrocytes by SHIP‐1 KO MSCs likely contributes to the greater vessel density and increased number of progenitor cells in the BM. We believe that osteoclasts may also play a role in the observed phenotype, which warrants further studies. We further speculate that excessive vessel invasion may deplete the osteoblastic progenitor pool of the perichondrium, resulting in thinner cortical bones, and augmented and disorganized trabeculae formation in the BM. The nature of the cellular and molecular mechanisms responsible for coupling angiogenesis and skeletal development remains poorly understood, but a primary driving force is tissue hypoxia (Schipani et al., 2001). Hypoxia environment of avascular mesenchymal tissues is critical for proliferation and differentiation of chondrocytes during endochondral bone formation (Robins et al., 2005; Schipani et al., 2001). Indeed, how osteochondroprogenitor cells behave under hypoxic conditions determines the outcome of skeletal development. The cell biological behavior of these progenitors is in part regulated by the activation of Akt. Since the 5′‐phosphatase SHIP‐1 modulates PI3K initiated signaling by limiting membrane recruitment and activation of Akt through both its substrate PI(3,4,5)P3 and its product PI(3,4)P2, the skeletal phenotype of SHIP‐1 KO mice should very much depend on how SHIP‐1 deficient osteochondroprogenitor behave under hypoxic conditions. Our data indicate that SHIP‐1 deficiency in the PαS MSCs leads to impaired HIF‐1α stabilization under the hypoxia condition. This result was further supported by experiments using a hypoxia‐mimicking chemical DFO. A reduced HIF‐1α protein level inhibits SHIP‐1 KO PαS MSC proliferation and possibly promotes hypertrophic differentiation through decreased Akt and Erk phosphorylation. Tissue specificity of the Prx1‐cre mouse model remains debatable but current literature support its application in targeting osteochondral progenitors (Elefteriou & Yang, 2011). Future studies cross‐breeding SHIP‐1 (flox/flox) mice with early chondrocyte specific Col2a1‐cre mice and hypertrophic chondrocyte specific Col10a1‐cre mice will allow us to define specific roles of SHIP‐1 in different stages of endochondral ossification.

Damaged or injured articular cartilage remains one of the most difficult tissue to heal. Tissue engineering techniques and cell‐based therapies including autologous chondrocyte implantation have not been proven successful in restoring the original articular cartilage structure and function in humans. One key question is how a hypertrophic phenotype of transplanted chondrocytes can be lastingly suppressed in regenerating tissue (Caldwell & Wang, 2015; Doran, 2015). Our current study has shed some light on the chondrocyte hypertrophy during endochondral ossification and suggests that SHIP‐1 may be a critical player regulating the cartilage formation process. It would be interesting to investigate whether overexpression of SHIP‐1 in MSCs or application of a small molecule agonist to enhance SHIP‐1 activity (Viernes, Choi, Kerr, & Chisholm, 2014) would delay the chondrocyte hypertrophic process in an autologous chondrocyte implantation osteoarthritis animal model.

## 5 CONCLUSION

In conclusion, the lipid phosphatase SHIP‐1 exerts a critical role in skeletal development and in establishing the BM microenvironment through controlling chondrocyte hypertrophy and the hypoxia response of the osteochondroprogenitors. Further studies using chondrocyte and hypertrophic chondrocyte specific targeted deletion of SHIP‐1 are warranted. Targeting SHIP‐1 expression in vivo has the potential to treat human skeletal dysplasia and adult abnormal fracture healing and may improve the outcome of cell‐based cartilage repair therapy.

## CONFLICT OF INTEREST

The authors declare that there are no conflict of interests.

## AUTHOR CONTRIBUTIONS

E.‐Y.S. and C.S.: collection and/or assembly of data, data analysis and interpretation, manuscript writing; K.Q.W., A.D., S.L., M.I., and T.S.: collection and/or assembly of data, data analysis and interpretation; P.M.D‐S. and A.M.R.: provision of study material, data analysis and interpretation, final approval of manuscript; O.D.L.: conception and design, collection and/or assembly of data, data analysis and interpretation, manuscript writing, final approval of manuscript. All the authors have read and approved the manuscript.

## ETHICS STATEMENT

Animal procedures followed protocols approved by the Institutional Animal Care and Use Committees at Rhode Island Hospital (Committee Nr. 0072‐14 and Nr. 5007‐17).

## DATA ACCESSIBILITY

The data that support the findings of this study are available from the corresponding author upon reasonable request.

## Supporting information

Supporting information

Supporting information

Supporting information

Supporting information
