# Supplementary figures and images for: Lipid phosphatase SHIP‐1 regulates chondrocyte hypertrophy and skeletal development
Source: J Cell Physiol. 2019 Jul 9;235(2):1425–37. doi: 10.1002/jcp.29063 (PMC6879780; doi:10.1002/jcp.29063)

## Slide 1
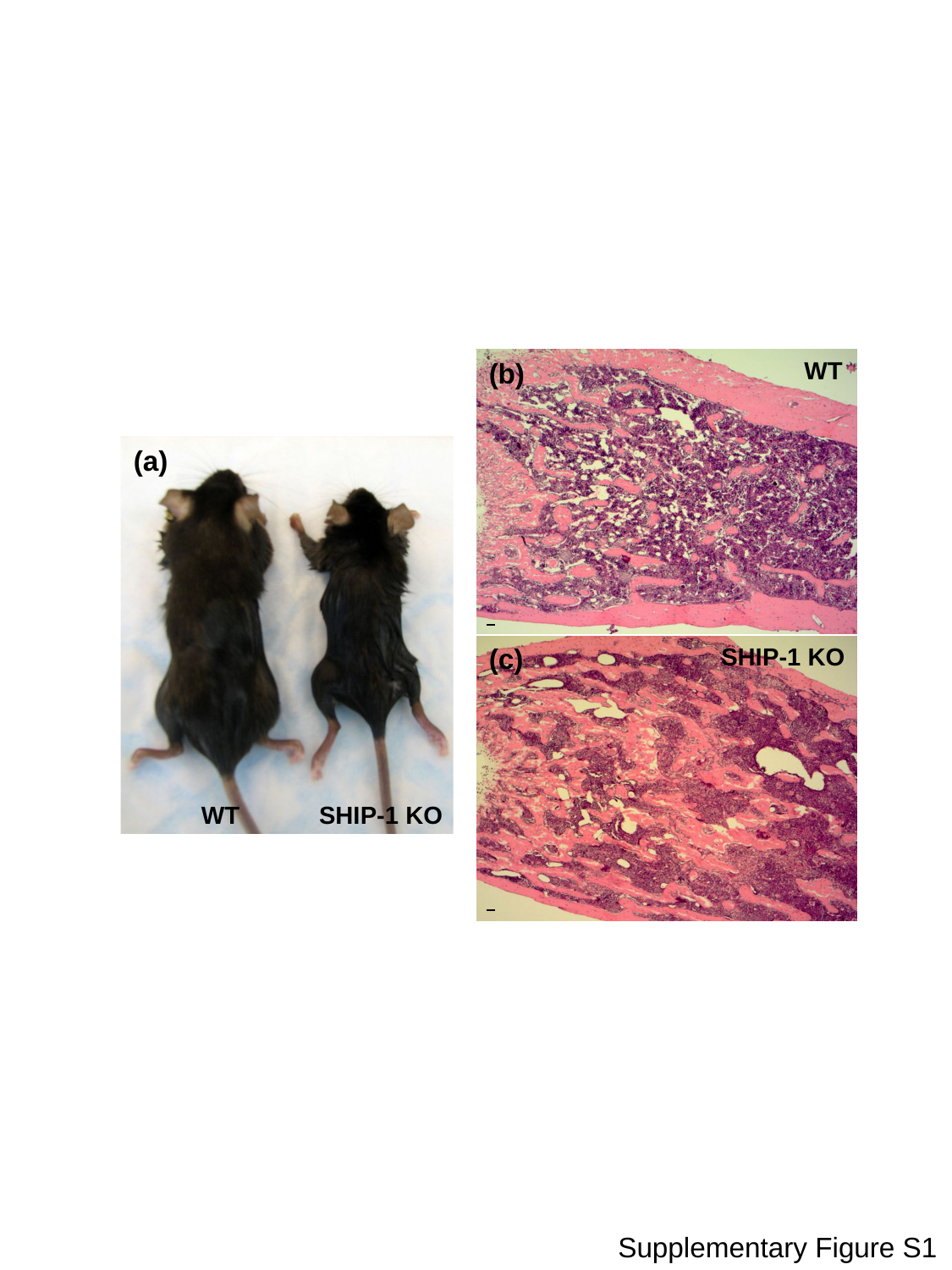

(b)
WT
(a)
(c)
SHIP-1 KO
WT
SHIP-1 KO
Supplementary Figure S1

Supplement: Supplementary file 1 — Supporting information [file JCP-235-1425-s001.pptx]

## Slide 1
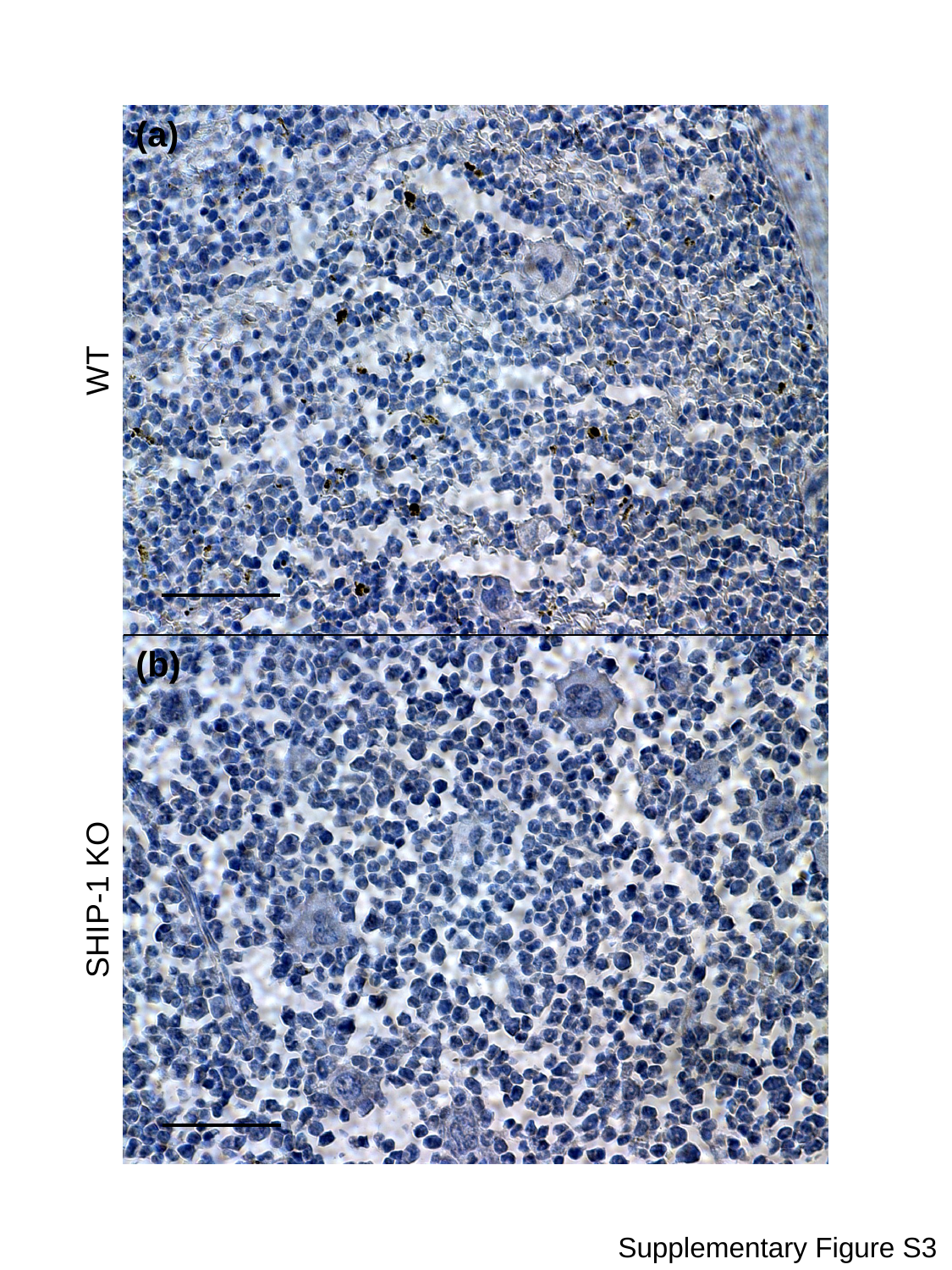

(a)
WT
(b)
SHIP-1 KO
Supplementary Figure S3

Supplement: Supplementary file 3 — Supporting information [file JCP-235-1425-s003.pptx]
