# Supplementary material for: Lipid phosphatase SHIP‐1 regulates chondrocyte hypertrophy and skeletal development
Source: J Cell Physiol. 2019 Jul 9;235(2):1425–37. doi: 10.1002/jcp.29063 (PMC6879780; doi:10.1002/jcp.29063)
Supplement: Supplementary file 2 — Supporting information [file JCP-235-1425-s002.pptx]

## Slide 1
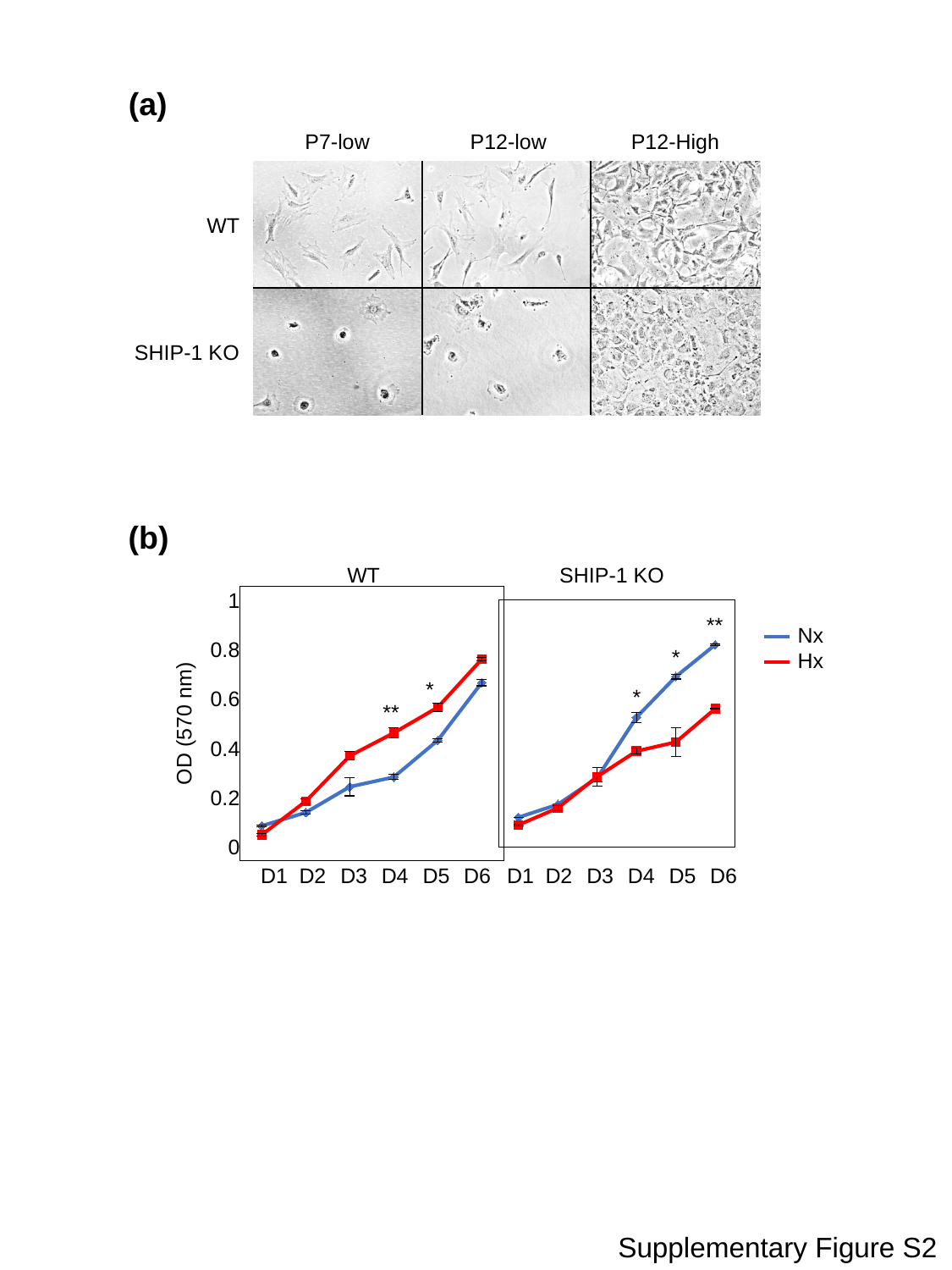

(a)
P7-low
P12-low
P12-High
WT
SHIP-1 KO
(b)
WT
SHIP-1 KO
### Chart
| Category | WT | WT-2% |
|---|---|---|
| D1 | 0.127 | 0.09450000000000004 |
| D2 | 0.17550000000000004 | 0.21650000000000005 |
| D3 | 0.2695 | 0.3820000000000001 |
| D4 | 0.3045000000000001 | 0.4655 |
| D5 | 0.4385000000000001 | 0.559 |
| D6 | 0.6490000000000002 | 0.7345 |
### Chart
| Category | KO | KO-2% |
|---|---|---|
| D1 | 0.11950000000000002 | 0.08900000000000004 |
| D2 | 0.17200000000000001 | 0.15750000000000006 |
| D3 | 0.2800000000000001 | 0.2845000000000001 |
| D4 | 0.525 | 0.3880000000000001 |
| D5 | 0.6900000000000002 | 0.4245000000000001 |
| D6 | 0.8190000000000002 | 0.56 |1
**
Nx
Hx
0.8
*
*
*
0.6
**
OD (570 nm)
0.4
0.2
0
D1
D2
D3
D4
D5
D6
D1
D2
D3
D4
D5
D6
Supplementary Figure S2
