# Supplementary material for: Lipid phosphatase SHIP‐1 regulates chondrocyte hypertrophy and skeletal development
Source: J Cell Physiol. 2019 Jul 9;235(2):1425–37. doi: 10.1002/jcp.29063 (PMC6879780; doi:10.1002/jcp.29063)
Supplement: Supplementary file 4 — Supporting information [file JCP-235-1425-s004.pptx]

## Slide 1
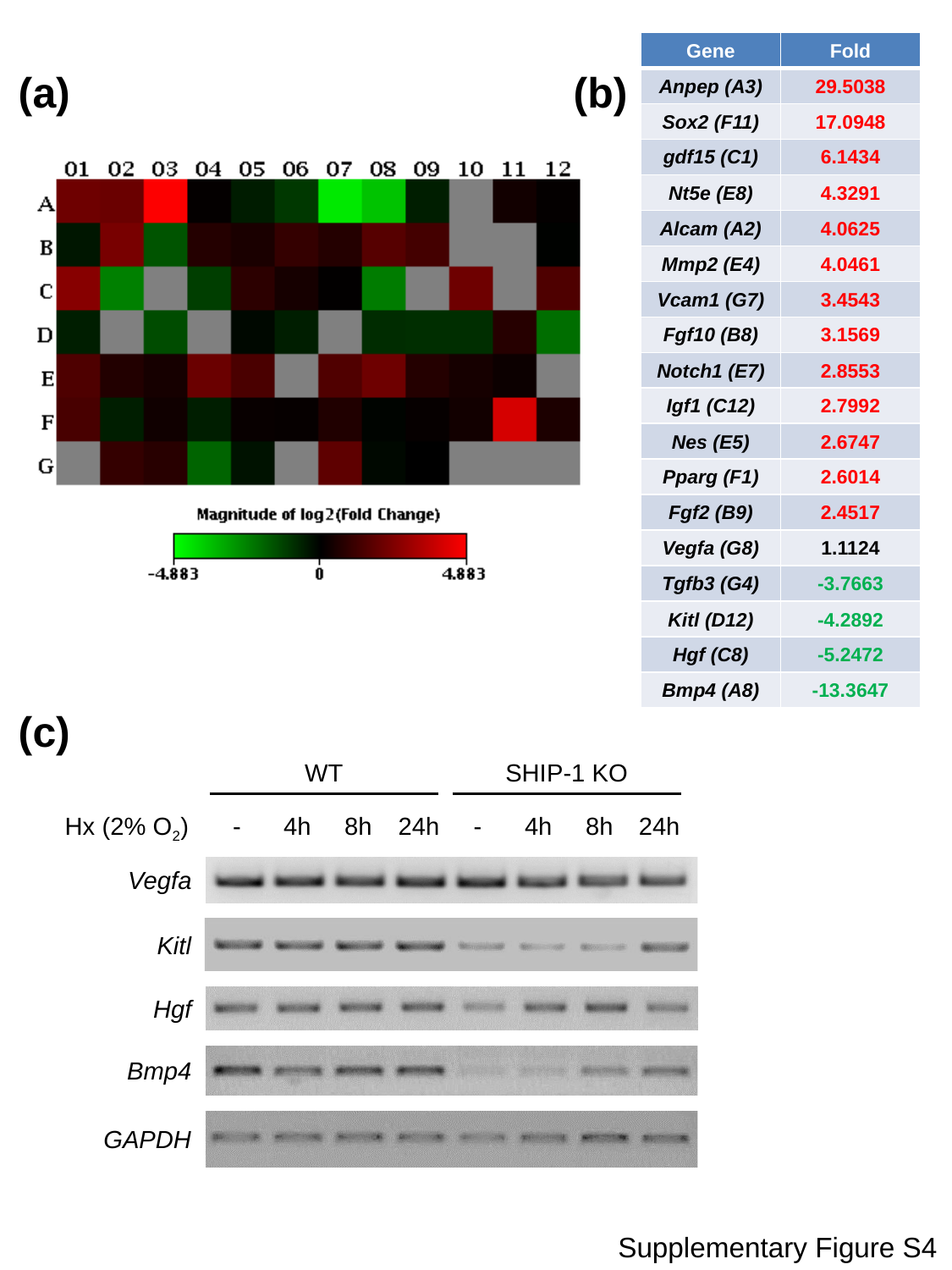

B
| Gene | Fold |
| --- | --- |
| Anpep (A3) | 29.5038 |
| Sox2 (F11) | 17.0948 |
| gdf15 (C1) | 6.1434 |
| Nt5e (E8) | 4.3291 |
| Alcam (A2) | 4.0625 |
| Mmp2 (E4) | 4.0461 |
| Vcam1 (G7) | 3.4543 |
| Fgf10 (B8) | 3.1569 |
| Notch1 (E7) | 2.8553 |
| Igf1 (C12) | 2.7992 |
| Nes (E5) | 2.6747 |
| Pparg (F1) | 2.6014 |
| Fgf2 (B9) | 2.4517 |
| Vegfa (G8) | 1.1124 |
| Tgfb3 (G4) | -3.7663 |
| Kitl (D12) | -4.2892 |
| Hgf (C8) | -5.2472 |
| Bmp4 (A8) | -13.3647 |
(a)
(b)
(c)
WT
SHIP-1 KO
Hx (2% O2)
-
4h
8h
24h
-
4h
8h
24h
Vegfa
Kitl
Hgf
Bmp4
GAPDH
Supplementary Figure S4
